# Age-related vs. disease-related: how perceptions of geriatric syndromes shape health-seeking behavior in older adults

**DOI:** 10.1186/s12877-025-06855-z

**Published:** 2026-02-02

**Authors:** Charlotte Kobus, Marlene Günther, Aline Schönenberg, Tino Prell

**Affiliations:** 1 Department of Geriatrics, Halle University Hospital, Ernst-Grube-Str. 40, Halle (Saale), 06120 Germany; 2 Department of Geriatrics, Jena University Hospital, Am Klinikum 1, Jena, 07747 Germany

**Keywords:** Geriatric syndromes, Health-seeking behavior, Age-related conditions, Disease-related conditions, Perceptions of aging, Views on aging

## Abstract

**Background:**

Geriatric syndromes (GS) describe complex health challenges in older patients. Understanding the perceptions of geriatric syndromes as age-related vs. disease-related can help to identify different patterns of health-seeking behavior (HSB) for GS among older adults.

**Methods:**

In this explorative, cross-sectional study, we investigate the prevalence and perception of various GS (falls, gait disturbance, pain, urinary incontinence, memory loss, depressive symptoms, loss of social contacts) in a geriatric cohort (*n* = 94, mean age 82.5 ± 5.29 years, 62.8% female). Variables included the presence of GS, their perceived impact, and whether they were viewed as age-related or disease-related (visual analogue scale). HSB was assessed based on whether patients sought medical consultation, received diagnostics, or underwent therapy for the GS. Additional variables included a comprehensive geriatric assessment, health literacy, locus of control, and views on aging.

**Results:**

The study found significant variations in HSB among different GS. Overall, 94.7% of the patients experienced at least one GS. The most frequently reported GS were falls (59.6%), gait problems (55.3%), and pain (51.1%). Incontinence (39.4%), falls (37.5%), and gait problems (32.7%) were considered the most relevant GS by the patients. Pain (89.6%), incontinence (72.7%), and gait problems (69.2%) were the primary reasons for seeking medical consultation, with therapeutic measures more commonly initiated for pain (70.8%) than for gait disorders (28.8%) and incontinence (33.3%). Symptoms of depression and memory loss received minimal medical attention.

Pain was predominantly perceived as disease-related, with corresponding higher HSB, whereas memory loss was often seen as age-related, with corresponding lower HSB. There was no significant association between GS ratings (age or disease-related) and sex, living situation, social support, education level, health literacy, or locus of control. However, a positive view on aging correlated with perceiving depressive symptoms and lack of energy as disease-related.

**Conclusion:**

This explorative study suggests that the individual perceptions of GS may impact the use of medical services. Future confirmatory studies are necessary to develop targeted interventions addressing these perceptions in order to improve the health behavior of older adults.

**Supplementary Information:**

The online version contains supplementary material available at 10.1186/s12877-025-06855-z.

## Background

Health-seeking behavior (HSB) refers to any action or inaction taken by individuals with health problems to find an appropriate remedy [1]. The HSB of older adults is influenced by a multitude of external and internal factors, including the burden and type of illness, socio-economic status, sex, and access to services. Furthermore, attitudes and personal beliefs about health and illness can also exert an influence on HSB [2]. These beliefs are shaped by a multitude of factors, including cultural, social, familial, and personal experiences. For example, individuals with more positive beliefs about aging tend to engage in more preventive healthcare, whereas those with negative beliefs about aging experience poorer health outcomes, engage in less physical activity, and use fewer preventive healthcare services [3–8].

The majority of existing studies on HSB in older adults have focused on specific conditions or diseases [9–11]. However, the impact of single diseases on health and well-being in older age is constrained. Rather, it is the coexistence of dysfunction across multiple systems that represents the predominant health challenge in older adults [12, 13]. These overarching age-related health challenges are commonly described as geriatric syndromes (GS). GS are highly prevalent with increasing age [14, 15] and typically refer to health issues such as immobility, instability, incontinence, cognitive decline, and depression. These conditions can have a substantial impact on the well-being and functional capacity of older individuals, often posing complex management dilemmas [16, 17]. Given the considerable impact of GS on individuals, families, and healthcare systems, there has been a notable increase in attention devoted to this area in recent years. While the majority of studies have concentrated on the perception of a single disease [18], there is a paucity of knowledge regarding the perception of GS. The Common Sense Model (CSM) of Self-Regulation provides a framework for understanding how individuals perceive, interpret, and respond to health threats, and how these perceptions influence their health behaviors and outcomes. The key dimensions of this model are identity (the label and symptoms associated with the illness), consequences (beliefs about the impact of the illness on one’s life), causes (beliefs about the factors that caused the illness), and control/cure (beliefs about the controllability and curability of the illness) [19, 20]. Notably, despite wide use of the CSM in single-disease contexts (e.g. diabetes, cardiovascular disease), no prior work has applied its core dimensions (identity, causes, consequences, control) to understand how older adults conceptualize and respond to multiple, co-occurring geriatric syndromes. This represents a clear gap in both theoretical development and the design of targeted interventions. By mapping each GS onto the CSM’s dimensions—how patients label (identity), interpret causes, anticipate consequences, and appraise controllability—we can derive specific hypotheses about which syndromes will be viewed as ‘age-related’ versus ‘disease-related’ and how these perceptions drive distinct health behaviour and help-seeking pathways. This can assist in elucidating health behaviors, offering insights into psychological adaptation to illness, and developing interventions targeting specific illness perceptions to enhance health outcomes [21–23]. In the context of aging research, it is also of interest to ascertain whether GS are regarded as age-related or as illness-related health issues, as this could influence health behavior.

In order to gain a deeper understanding of the personal perspective on GS as age- or disease-related phenomena, we conducted further data collection on general views on aging (VoA), locus of control, and health literacy [24, 25]. The concept of locus of control delineates individuals’ beliefs about their influence over life events. These beliefs can be internal, involving personal agency, or external, attributing events to fate, luck, or powerful others [26]. VoA are used to describe individuals’ perceptions and attitudes towards their own aging process and the concept of aging in general. These perceptions encompass beliefs about the physical, social, and psychological changes that accompany the aging process. VoA are of paramount importance for elucidating health outcomes in older adults [27]. Individuals who perceive aging in a positive light, such as viewing it as a period of continuous growth, have been found to engage in proactive health behaviors [28] and have better physical and mental health [29].

A deeper understanding of how older adults attribute GS to aging versus disease is a necessary first step in the design of targeted interventions—for example, educational modules or decision aids—that explicitly reframe age-attributed symptoms as treatable. Such interventions can then be empirically tested for their ability to foster more positive views of aging and, ultimately, to improve health behaviors, outcomes, and quality of life.

However, it remains unknown (1) whether CSM-derived illness perceptions align with a continuum from ‘age’ to ‘disease’ attributions for GS and (2) how these attributions predict actual health-seeking behaviours. To address this, we posed the following research questions: (1) What are the prevalence rates of key GS in a hospitalized geriatric cohort? (2) How do older adults’ perceptions—framed by CSM constructs—place each GS on an age–disease continuum? (3) To what extent do these age–disease attributions predict differences in health-seeking behaviours across syndromes? These data could inform the development of interventions to enhance health behavior in older adults [30].

## Methods

### Study design

This prospective, cross-sectional observational study was conducted between March 2023 and December 2023 in the Centre for Geriatrics in Southern Saxony-Anhalt (Zentrum für Altersmedizin im südlichen Sachsen-Anhalt, ZASSA). We included geriatric patients that received geriatric early rehabilitative complex treatment, which is a specialized treatment approach for older hospitalized patients with acute illnesses or injuries, recorded under the Operations and Procedures Key (OPS) system 8-550. This treatment involves a multidisciplinary team including geriatricians, nurses, physiotherapists, occupational therapists, speech therapists, social workers, psychologist, and other professionals. We excluded patients who were not able to provide valid self-report due to severe health problems and patients with delirium or severe dementia. All patients gave written informed consent. Local ethics committee of Halle University Hospital approved the study.

Overall, 323 patients were treated in the geriatric early rehabilitative complex treatment during the study period. As shown in Supplement Fig. 1, data from 94 patients were used for analyses.

### Variables of interest

#### Geriatric syndromes

Patients were asked by trained study staff if the following GS were present prior to hospitalization: falls, gait disturbance, pain, incontinence, memory loss, depressive symptoms, and loss of social contacts. We did not ask directly about depression because of the negative stigma attached to it [31]. Instead, we asked about three major depressive symptoms (loss of interest for hobbies, lack of joy, and lack of motivation or energy).

Additionally we asked, which GS each patient considered as most important/relevant.

To assess whether patients attributed a GD to age or disease, we used a visual analogue scale presenting a slider-style response format ranging from 0 = disease to 100 = age. Participants marked the point on the line that best represented their assessment of what proportion a GS was caused by disease or advancing age. This numeric variable is named VAS-GS.

#### HSB - health seeking behavior

If the GS was present, we asked if they (1) ever contacted a doctor about it, (2) ever had any medical examination about it, (3) ever had therapy targeting it. If they had never contacted/had no examination/had no therapy regarding the GS, we asked them in an open-ended question what prevented them from doing so.

#### Covariates

To measure health literacy, we used the item from the Survey of Health, Aging and Retirement in Europe (SHARE): “How often do you need someone to help you read instructions, pamphlets, or other written materials from your doctor or pharmacy?” [32, 33], which is rated on a Likert scale (always, often, sometimes, rarely, and never).

To measure internal and external control-beliefs, we used the German Internal–External Locus of Control Short Scale–4 (IE-4) scale (Skala Internale-Externale-Kontrollüberzeugung-4 (IE-4)). The scale consist of four items, two each for internal and external control-beliefs on a 5-point Likert scale. Higher scores indicate higher internal or external Locus of control [34, 35].

To assess VoA, we used a questionnaire on subjective ageing (Bereichsspezifisches subjektives Alterserleben) from the German Aging Survey (DEAS), a large population-based study of Germans aged 40 and older. It covers views on age-related changes in four distinct subscales: Physical losses decline (refers to the view of aging as accompanied by physical losses), social losses (e.g. no longer being needed by others or decreased respect), continuous growth (implies that aging is also seen as a time of ongoing personal development), and self-awareness (gains). The participants rated the items on a scale ranging from 1 (definitely true) to 4 (definitely false), and we reverse scored the items for the analyses [36].

For all patients, we collected sociodemographic and medical parameters as well as the comprehensive geriatric assessment to characterize the cohort in detail. The geriatric assessment is performed by trained hospital staff as part of everyday clinical care:

- age (years, metric), sex (male/female, dichotomous),
- marital state (single/married/widowed/separated, multinominal),
- education level (no degree/elementary school/secondary school/high school diploma), multinominal),
- living situation (alone/with partner or family/assisted living/nursing home/others, multinominal),
- care level (no care level/care level 1–4, ordinal), need of nursing service (Yes/No/No but with help from relatives, multinominal).
- main diagnosis.
- Screening by Lachs consists of 15 dichotomous questions regarding problems with eyesight, hearing ability, mobility, continence, nutrition, activity, mood, social support etc. One point is given for every malfunction [37].
- Barthel Index [38, 39]: Describes functional level for ten daily tasks (including eating, personal hygiene, mobility, continence).
- Mobility measured by Tinetti-Test assesses risk of falling by testing gait and balance. Maximum number of points: 28 [40].
- Cognitive function measured by Mini-Mental state examination (MMSE) screens orientation, memory, attention, calculating and language ability with a maximum number of 30 points [41]; of note, the MMSE was performed as part of the clinical routine assessment by hospital staff in general patient care;
- Geriatric depression scale (GDS): dichotomous questionnaire with 15 items assessing emotion and behavior towards life with maximum number of 15 points [42, 43].

### Statistical analyses

All calculations were performed using SPSS 28.0. The cohort was characterized using descriptive statistics (Mean (M), Standard Deviation (SD)). The Shapiro-Wilk test was employed to test for normal distribution. The association between VAS-GS and other variables was tested with group comparison (t-test or U-test) or Spearman correlation. The significance level was 0.01. The exploratory items were noted by trained staff during face-to-face interviews (CK, MG) and sorted into categories by one of the researchers (CK). Reasons for not addressing issues with physician were categorized into (1) symptoms are consequences of normal ageing (2) perception of relevance or insignificance of symptoms, (3) lack of confidence or time to visit doctor, (4) assumptions about doctor’s role and abilities, (5) others. All exploratory items are reported in terms of category quantity or percentages with the aim to further substantiate the quantitative data and gather a deeper understanding of the patients’ experiences.

## Results

### Description of the cohort

Table 1 provides a summarized description of the cohort. The final cohort studied had a mean age of 82.5 years (SD = 5.3 years), and 62.8% were female. The most common causes of hospitalization were trauma (40.4%) and cardiovascular disease (23.4%) (Supplement Fig. 2). The majority of participants were widowed or divorced (68.1%) and lived alone (55.3%) (Supplement Table 1). Values for VoA, health literacy, and locus of controls are given in Supplement Table 2. On average, each participant was taking 8.5 (SD = 4.1) different medications daily. Comprehensive geriatric assessments revealed that 54.3% of participants had severe limitations according to the Barthel index, while 33% had moderate to severe limitations in activities of daily living. Nursing services were used by 41.9% of the cohort and an additional 9.7% relied on family members for support. In addition, 56.2% reported frequent pain and 70.2% of the cohort reported varying degrees of sadness and depression. Detailed characteristics from geriatric assessments are provided in Supplement Table 1.

**Table 1:** Comprehensive characteristics of the entire cohort (*N* = 94)

Female / Male, *n* (%)	59 (62.8) / 35 (37.2)
Age (years), mean ± SD	82.5 ± 5.29
Number of medication per day, mean ± SD	8.49 ± 4.07
Numer of diagnoses	13.81 ± 4.70
Body-mass-index (kg/m2), mean ± SD	27.13 ± 6.51
Hand grip strength (bar), mean ± SD	0.37 ± 0,18
Barthel index sum score, mean ± SD	34.73 ± 12.71
Tinetti test sum score, mean ± SD	12.81 ± 4.87
Mini-Mental-Status-Examination, mean ± SD	27.02 ± 2.65
Geriatric Depression Scale, mean ± SD	2.82 ± 2.87
Lachs test sum score, mean ± SD	6.69 ± 2.10

### Health seeking behavior because of geriatric problems

**Fig. 1:**
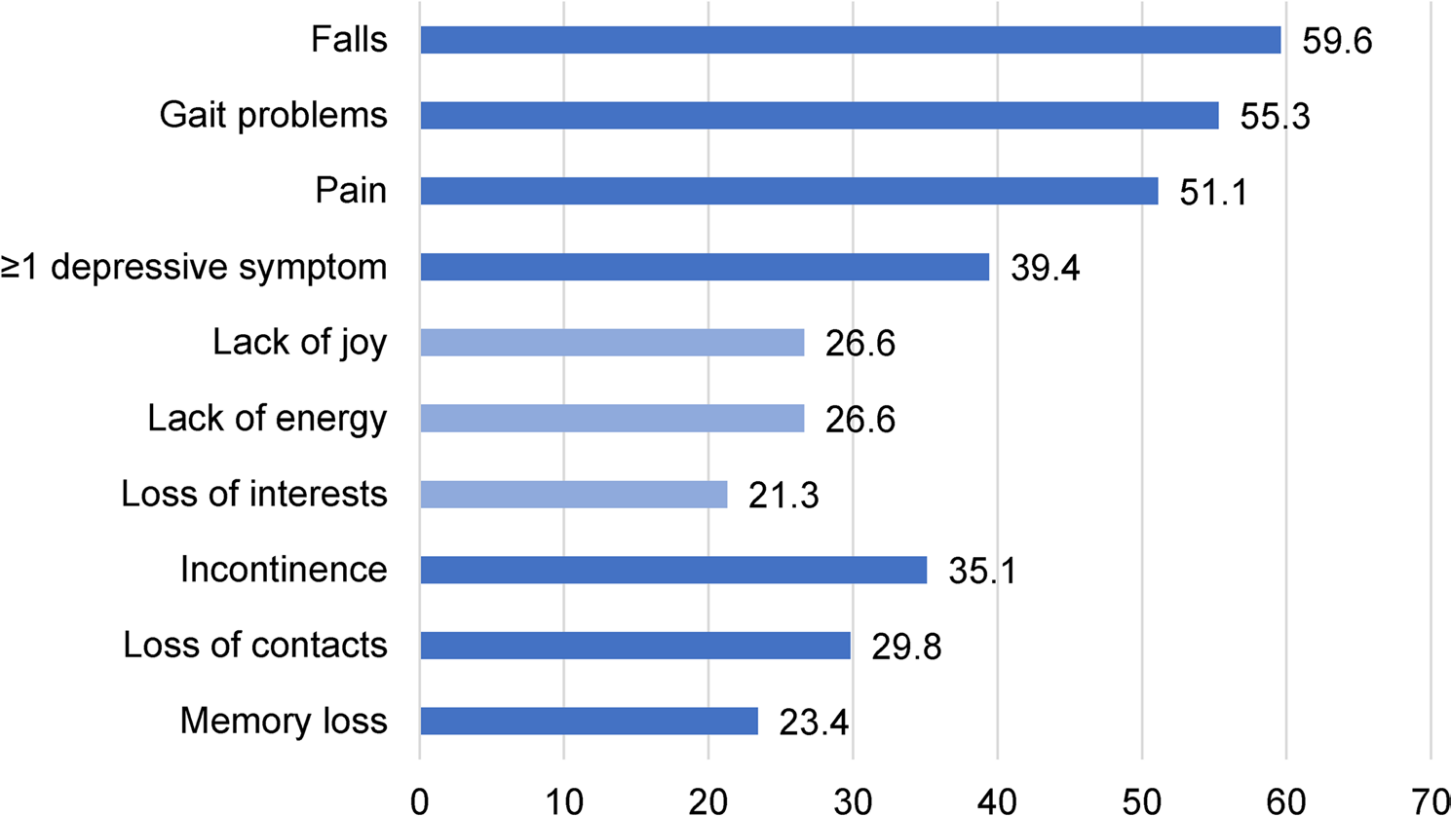
Prevalence of geriatric syndromes (%, *N* = 94)

Overall, 94.7% of the patients experienced any GS prior to being hospitalized. The three most frequently reported GS were falls (59.6%), gait problems (55.3%), and pain (51.1%) (Fig. 1). Among the GSs, incontinence (39.4%), falls (37.5%), and gait problems (32.7%) were regarded as most important for the patients.

The primary reasons for seeking medical consultation were pain (89.6%), incontinence (72.7%), and gait problems (69.2%). However, therapeutic measures were initiated more frequently for pain (70.8%) than for gait disorders (28.8%) and incontinence (33.3%). Symptoms of depression and memory loss received minimal medical attention, as shown in Table 2.

**Table 2:** Geriatric syndromes and health care utilization

Geriatric syndromes	If geriatric syndrome was present…			
	…selected as most relevant, %	…physician consulted, %	…medical diagnostics arranged, %	…therapy arranged, %
Falls	37.5	53.6	35.7	12.5
Gait problems	32.7	69.2	40.4	28.8
Pain	29.2	89.6	72.9	70.8
Incontinence	39.4	72.7	33.3	33.3
Memory loss	18.2	36.4	13.6	4.5
Loss of contacts	25	14.3	0	0
≥ 1 depressive symptom:	21	21.6	2.7	8.1
Lack of energy	20	24	0	0.08
Lack of joy	8	24	4	0.08
Loss of interests	5	15	0	0.05

For each geriatric syndrome (GS), percentages refer to the subgroup of patients in whom that syndrome was present (denominator = number of patients reporting the GS). “Selected as most relevant” indicates the proportion of those patients who rated the GS as their single most important health concern. “Physician consulted,” “Medical diagnostics arranged,” and “Therapy arranged” show the percentage of patients with the GS who ever (1) contacted a physician about it, (2) underwent any diagnostic evaluation, or (3) received a targeted therapeutic intervention, respectively. The varying denominators across syndromes reflect differences in prevalence

*GS* geriatric syndrome

The most frequent GS for which a physician was not consulted were loss of contact (78.6%), depressive symptoms (78.4%), memory problems (54.5%), and falls (46.4%). In these cases we asked an open-ended question why the patients did not seek medical consultation for the GS (Table 3). For falls, the primary reason cited was the perception that the symptoms were irrelevant or unimportant, accounting for 50% of the cases. Similarly, for gait disturbances, 35.71% of respondents felt the symptoms were not significant enough to warrant a doctor’s visit. For pain, the main reason was assumptions about the role and capabilities of the physician, with 50% of respondents believing that a doctor would not be able to help. For urinary incontinence, the primary reason was a lack of trust or time for doctor visits, cited by 44.44% of the respondents.

**Table 3:** Reasons not to consult physician for a geriatric syndrome

	Symptoms are consequences of normal ageing % (*n*)	Perception of relevance or insignificance of symptoms % (*n*)	Lack of confidence or time to visit doctor % (*n*)	Assumptions about doctor’s role and abilities % (*n*)	Others % (*n*)
Falls (*n* = 24)	0 (0)	50 (12)	29.17 (7)	12.5 (3)	8.33 (2)
Gait problems (*n* = 14)	0 (0)	35.71 (5)	14.29 (2)	28.57 (4)	21.43 (3)
Pain (*n* = 4)	0 (0)	25 (1)	25 (1)	50 (2)	0 (0)
Incontinence (*n* = 9)	22.22 (2)	11.11 (1)	44.44 (4)	0 (0)	22.22 (1)
Memory loss (*n* = 11)	45.45 (5)	18.18 (2)	36.36 (4)	0 (0)	9.09 (1)
Loss of contacts (*n* = 21)	0 (0)	14.29 (3)	28.57 (6)	38.1 (8)	14.29 (3)
Loss of interests (*n* = 11)	0 (0)	27.27 (3)	18.18 (2)	36.36 (4)	18.18 (2)
Lack of joy (*n* = 18)	0 (0)	16.67 (3)	33.33 (6)	33.33 (6)	22.22 (4)
Lack of energy (*n* = 18)	16.67 (3)	11.11 (2)	27.78 (5)	27.78 (5)	16.67 (3)

Memory problems were mostly perceived as a normal part of aging, which led 45.45% of respondents to refrain from consulting a physician. For reduced social contacts, the main reason was assumptions about the physician’s role (i.e., physician is not responsible for this issue), mentioned by 38.1% of respondents. In the case of loss of interest, 36.36% of respondents did not seek medical advice due to assumptions about the physician’s capabilities. For feelings of joylessness, the reasons were equally split between a lack of trust or time for doctor visits and assumptions about the physician’s role, each accounting for 33.33% of the cases. Lastly, for fatigue, the primary reasons were equally divided between lack of trust or time for doctor visits and assumptions about the physician’s capabilities, each cited by 27.78% of the respondents. Overall, the main reasons for not consulting a physician varied across different problems but commonly included the perception of symptoms as irrelevant or unimportant, a lack of trust or time for doctor visits, and assumptions about the physician’s role and capabilities (refer to Table 3).

### Classification of GS as age-related or disease-related

**Fig. 2:**
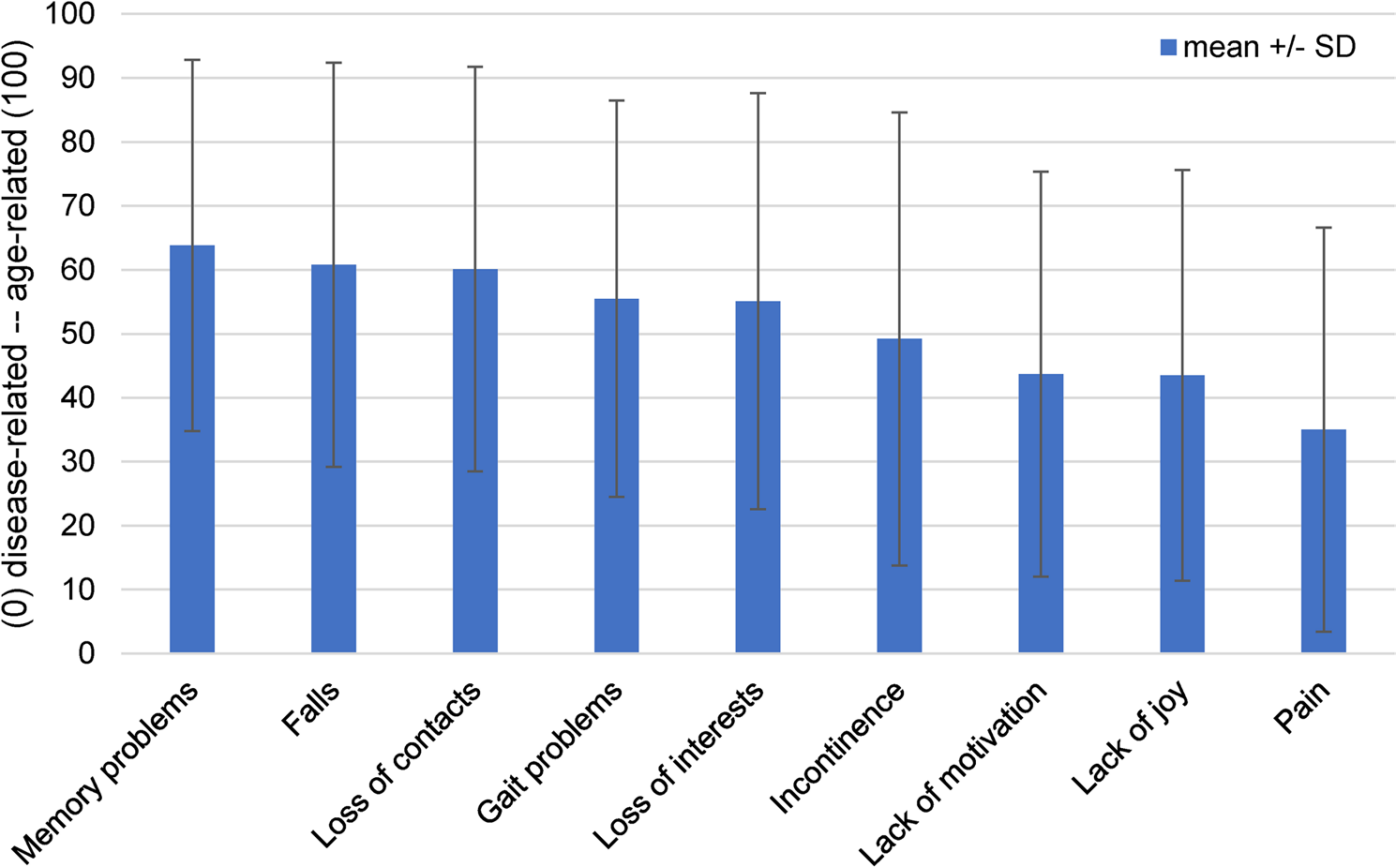
Classification of geriatric syndromes as age-related or disease-related

To investigate participants’ perceptions of the differentiation between illness and aging, they were asked to rate their GS on a scale from 0 (related to illness) to 100 (related to age). Pain was mostly rated as related to illness (M = 35; SD = 31.6). Conversely, memory loss was the GS most strongly associated with age (M = 63.81; SD = 29.007) (see Fig. 2).

Rating a GS as age-related or disease-related was not significantly associated with sex, living situation, social support, education level, health literacy, and locus of control (all *p* > 0.01). It did also not correlate with the VoA subscales physical decline, social losses (e.g. no longer being needed by others or decreased respect), and self-awareness (gains). However, the VoA subscale continuous growth correlated negatively with VAS lack of joy (*r*=-0.298, *p* = 0.006), lack of interests (*r*=-0.298, *p* = 0.005), lack of energy (*r*=-0.329, *p* = 0.002), and loss of contacts (*r*=-0.371, *p* = 0.001). This indicates that people with positive VoA (i.e. thinking that aging provides opportunity for continuous growth) are more likely to regard these symptoms as being disease-related instead of age-related.

## Discussion

The study analyzed healthcare utilization for geriatric concerns and found that falls, gait problems, and pain were the most commonly reported issues. Incontinence, falls, and gait problems were identified as the most relevant issues by patients. Pain was the primary reason for seeking medical consultation, with therapeutic measures initiated more frequently for pain compared to gait disorders and incontinence. In contrast, depressive symptoms and memory loss received minimal medical attention. Reasons for not seeking medical advice included beliefs that it would not make a difference, attributing symptoms to aging, or not considering the issue significant. Participants perceived pain as more related to illness and memory loss as more related to age.

In line with an earlier study, the majority of our patients had at least one GS [15]. In our study, 51.1% of participants experienced chronic pain, which is consistent with the 40–80% prevalence reported in older populations in other studies [44–46]. Nearly 60% reported occasional falls or near falls, and 55.3% had gait disturbances. This aligns with the observation that trauma was a common reason for hospitalization in our cohort. Therefore, the prevalence of falls is higher in our cohort than in previous studies [47, 48]. Approximately 35.1% reported urinary incontinence, which is slightly higher than the 27.5% prevalence previously found in older Germans [48]. Approximately 23% of our subjects reported memory problems, similar to the 27% found earlier by Gaertner, Scheidt-Nave [48]. Of note, we excluded patients with severe dementia in our study to ensure valid self-report. In our study, approximately 30% reported loss of contact. This is in line with a systematic review estimating prevalence of loneliness and social isolation amongst the community-dwelling and institutionalized oldest old [49]. Overall, our cohort can be considered representative in terms of age-related geriatric vulnerability.

The findings of our study indicate that pain and depressive symptoms (e.g., lack of motivation, lack of joy) were predominantly perceived as disease-related, whereas memory loss, falls, and loss of contact were more commonly regarded as age-related. Gait problems, loss of interest, and incontinence were perceived as being on a continuum between age-related and disease-related factors. Our findings indicate that pain was primarily viewed as disease-related, a result that is consistent with the findings of Cornally and McCarthy [50]. In their study, the researchers reported that elderly individuals with chronic pain exhibited slightly higher age-related beliefs than organic beliefs about the cause of pain. Memory problems were frequently perceived as age-related, aligning with the findings of an earlier study [51]. While falls were predominantly associated with age, the relationship between gait problems and age or disease was inconclusive, as no supporting studies were identified.

Incontinence was not explicitly attributed to either age or disease. Other studies of select cohorts with urinary incontinence have demonstrated that incontinence is often perceived as a normative aspect of aging [52]. In a secondary analysis of data from older Canadian women with urinary incontinence, more than two-thirds of the participants indicated that they believed incontinence to be a normal part of the aging process [53].

Furthermore, these classifications of GS as age- or disease-related were not associated with health literacy, locus of control, age, or sex. However, we did observe an association between mental GS and VoA. Individuals who perceive aging as an opportunity for continuous growth are more likely to view depressive symptoms as being disease-related rather than age-related. The differences in the classifications of GS as age- or disease-related may reflect differing beliefs about aging: some view aging as a fixed, inevitable process (essentialist view), while others see it as flexible and modifiable (nonessentialist view). Those with a nonessentialist perspective tend to feel younger than their chronological age and can better mitigate negative stereotypes and low social status impacts [54]. Viewing ageing as fixed is associated with feeling threatened by aging and perceiving a limited future. While much research has explored attitudes towards aging and age-related stereotypes, there is a gap in validating measures that specifically capture beliefs about the malleability of the aging process [55].

A further objective of our study was to examine the HSB in relation to different GS. HSB is a complex process that is influenced by a number of factors, including perceived self-efficacy, positive outcome expectancies, and risk perception [34, 56]. Although there is an overall increase in the utilization of medical services with increasing age, our findings indicate that there is a notable underutilization for certain syndromes experienced by patients, particularly for psychosocial and memory problems. We observed discrepancies in the rationale behind the decision to seek or forego medical care for different GS. For instance, medical treatment was less frequently sought for memory problems. Jiao, Chang [51] identified that attributing memory problems as a medical condition represents a crucial initial step in the process of seeking medical care for these issues. The majority of our participants attributed memory problems to age. Other studies have indicated that elderly individuals tend to attribute memory problems to age and therefore refrain from seeking assistance [51, 57, 58].

The most prevalent reasons for the avoidance of professional assistance for psychosocial issues were the preconceptions about the scope and capabilities of medical practitioners and the dearth of self-assurance or time to schedule an appointment. These findings are corroborated by other studies [59] indicating that individuals aged 65 years and above are the least likely to utilize psychiatric or psychotherapeutic medical services [60, 61]. This is particularly significant when considering the fact that the prevalence of depression increases with age [62].

Falls and gait problems were identified as significant health concerns in our cohort, with a high proportion of individuals seeking medical attention due to these issues. Nevertheless, in accordance with the findings of an earlier study, it was observed that between 30% and 50% of individuals did not pursue medical consultation due to concerns related to falls or gait problems [63].

It is noteworthy that the most common reason for not consulting a physician was the perception that falls or gait problems were not a significant issue to communicate to a medical professional. This may indicate that the harmfulness of falling and having gait problems is underrated. Older individuals have the highest risk of death or serious injuries caused by falling [64], yet there appears to be a lack of awareness of this fact in older patients. This is detrimental, as scientific evidence suggests that prevention is more effective than treatment in reducing the burden of falls [65].

The majority of respondents indicated that pain was primarily a disease-related problem and the most common reason for consulting a physician. This finding is consistent with those of other studies, which also suggest that pain is predominantly perceived as a symptom indicative of underlying bodily dysfunction. Furthermore, the attribution of pain as an organic cause has been identified as a significant predictor of help-seeking behavior [50, 66, 67]. The high utilization of medical services may be mediated by the duration and intensity of pain, as well as the disability caused by pain. These factors have been demonstrated to positively influence health-seeking behavior [68, 69].

The majority of participants (72.7%) who were experiencing urinary incontinence had discussed the issue with their physician. This figure is considerably higher than that reported in other surveys, which suggest that only approximately 30% of women experiencing UI will consult with their physician [70]. The observed differences in consultation rates may be attributed to factors such as age, duration of incontinence, and the severity of symptoms. In our cohort, the primary reasons for not seeking medical consultation were a lack of confidence or time to visit a doctor. In accordance with our findings, age-stereotypes about incontinence can also serve as a rationale for not seeking assistance, as individuals may perceive it as a normal aspect of aging devoid of substantial therapeutic alternatives [53, 70–72]. This suggests that incontinence, which is often perceived as an age-related condition, can also cause significant distress. This is a positive predictor for help-seeking behavior [73]. Participants explained how incontinence can lead to the loss of social contacts due to a loss of range of motion and a sense of stigma. This is related to other research indicating that perceived stigmata are positively associated with health-seeking behaviors [74, 75].

Our findings suggest that healthcare professionals can substantially improve timely help-seeking among older adults by addressing attributional biases in the way geriatric syndromes are presented and managed. First, clinicians should consciously frame certain syndromes—particularly those associated with mobility, balance, or sensory function—not as inevitable consequences of aging but as treatable medical conditions. By explicitly labeling falls risk, gait disturbances, or memory problems as abnormalities warranting clinical attention, providers can prompt more rapid patient engagement with diagnostic and therapeutic services. Second, to counteract the tendency of older adults to underreport or delay seeking care for symptoms they perceive as “just age,” routine screening should be embedded within annual geriatric assessments. Finally, shared decision-making tools that reframe age-attributed symptoms as modifiable can further enhance uptake of recommended interventions. Brief, easily understood decision aids —illustrating, for example, how balance training reduces fall risk—can shift perceptions of personal control and encourage acceptance of treatment. Together, these strategies leverage our understanding of illness representations to align clinical communication and practice with the CSM, ultimately promoting more effective, patient-centered care in geriatric populations.

Our explorative study is not free of limitations. The study’s small sample size limits its generalizability, necessitating larger studies to confirm the findings. The cross-sectional design captures data at one point in time, preventing causal inferences, and understanding the exact association between GS and HSB requires longitudinal studies to explore relationships over time. Our reliance on self-reported measures introduces potential recall and social desirability biases, which may be particularly pronounced for syndromes subject to stigma—such as memory loss and depressive symptoms—and could help explain why these conditions elicited less medical attention in our sample. Individuals may underreport cognitive or mood disturbances due to fears of losing autonomy or being labeled “demented,” thereby attenuating observed help-seeking intentions. Additionally, excluding patients with severe dementia or delirium may underrepresent the most vulnerable older adults, affecting the study’s comprehensiveness. Conducted in Southern Saxony-Anhalt, Germany, the findings may not be applicable to other cultural or healthcare contexts. The study does not deeply explore how variations in health literacy and cognitive function interact with perceptions and behaviors, warranting further investigation. Factors like socioeconomic status and previous healthcare experiences were not exhaustively controlled, potentially confounding the results. These limitations highlight the need for larger, more diverse, and longitudinal studies with objective measures to validate and extend the findings.

## Conclusion

Our study demonstrates substantial differences in HSB among older adults: symptoms such as pain, incontinence, and gait disturbances drove the highest rates of medical consultation, while memory loss, social isolation, and depressive symptoms were rarely reported. This pattern reflects a common tendency to interpret certain symptoms as “normal aging” rather than as medical conditions amenable to treatment. To mitigate these discrepancies, clinicians should adopt targeted strategies: first, they should reframe their communication by explicitly labeling cognitive and mood symptoms as treatable medical conditions; second, they should incorporate routine screening for mood and cognitive changes into annual geriatric assessments instead of relying solely on patient self-report; and third, they can deploy brief decision aids that clearly illustrate the benefits and controllability of interventions.

Looking ahead, research should move beyond descriptive analyses and rigorously test interventions designed to reshape illness perceptions. Educational workshops, digital decision-support tools, and other tailored programs should be evaluated for their impact on actual healthcare utilization and patient well-being over time. By translating the CSM into practical, evidence-based interventions, we can meaningfully improve the timeliness and effectiveness of geriatric care.

## Supplementary Information

Supplementary Material 1.

## Data Availability

The datasets used and/or analysed during the current study are available from the corresponding author on reasonable request.
